# Systematic dysregulation of immune-related alternative polyadenylation in systemic lupus erythematosus contributes to patient stratification

**DOI:** 10.1016/j.jtauto.2026.100378

**Published:** 2026-06-05

**Authors:** Ziyi Chen, Jingjing Shi, Yusuo Meng, Sidong Xiong, Hang Ruan

**Affiliations:** a The Fourth Affiliated Hospital of Soochow University, Institutes of Biology and Medical Sciences, Suzhou Medical College of Soochow University, Soochow University, Suzhou, 215123, China; b Jiangsu Key Laboratory of Infection and Immunity, Soochow University, Suzhou, 215123, China; c Biomedical Basic Research Center (BBRC) of Jiangsu, Soochow University, Suzhou, Jiangsu, 215123, China; d MOE Key Laboratory of Geriatric Diseases and Immunology, Suzhou Medical College of Soochow University, Soochow University, Suzhou, 215123, China; e Cancer Institute, Suzhou Medical College, Soochow University, Suzhou, Jiangsu, 215123, China

**Keywords:** Systemic lupus erythematosus, Alternative polyadenylation, 3′UTR lengthening, Stratification

## Abstract

**Background:**

Alternative polyadenylation (APA) is a common post-transcriptional gene regulatory mechanism. Although dysregulated APA is associated with various human diseases, its role in Systemic Lupus Erythematosus (SLE) remains largely unexplored. We systematically characterized immune-related APA alterations in SLE and investigated their potential for clinically useful patient stratification.

**Methods:**

We integrated RNA-seq data from multiple SLE cohorts involving peripheral blood samples and quantified APA using the DaPars2. We developed a scoring framework to identify key immune-related APA events (ImmAPA) and their utility for grouping SLE patients. And we used the Connectivity Map to explore potential differences in drug response across ImmAPA groups and examined these associations in an independent SLE cohort treated with an immunosuppressive drug.

**Results:**

SLE patients exhibited a recurrent trend in lengthening of the 3′ untranslated region (3′UTR) across cohorts. Twenty key APA regulators showed enhanced associations with these changes in SLE patients. The ImmAPA score can group patients into ImmAPA-high and ImmAPA-low subtypes, reflects activation states, and correlates with disease severity. The ImmAPA score captured complementary transcriptomic variation compared with interferon signature. CMap analysis predicted drugs that could reverse these APA changes, and was supported by follow-up analysis.

**Conclusions:**

Our study reveals a recurrent and widespread shift toward 3′UTR lengthening in SLE at peripheral blood transcriptome level. The ImmAPA framework provides a new perspective for SLE patient stratification and offers insights into precision therapeutic strategies in SLE.

## Highlights

Global 3′UTR lengthening is the primary APA remodeling pattern in SLE, as indicated by multi-cohort bulk RNA-seq profiling.

ImmPathAPAScore, a pathway-level framework, identifies a consistent set of immune-related APA events (ImmAPA; n = 202).

An ImmAPA-based ImmAPA score stratifies patients into ImmAPA-high or ImmAPA-low states within independent cohorts.

Candidate compounds are identified from transcriptomic reversal analysis and preliminarily explored in an external MMF follow-up cohort.

## 1 Introduction

Systemic lupus erythematosus (SLE) is a complex autoimmune disease affecting multiple organ systems and resulting in significant clinical heterogeneity [1]. SLE presents significant challenges in clinical practice due to substantial variability in treatment efficacy and high patient heterogeneity [2]. More precise patient stratification methods and biomarkers are needed to reflect underlying molecular heterogeneity, thereby facilitating the implementation of personalized treatment strategies. Large-scale transcriptomic profiling and modular analyses have established that interferon (IFN) centered transcriptional programs, including type I and type II IFN signatures, are highly stable in SLE and can be used to stratify patients [3]. Hubbard et al. constructed novel disease phenotypes using scores derived from immune cell and inflammatory pathway modules. The results indicate that distinct phenotypes correspond to different disease activities and clinical characteristics [4].

In a post hoc analysis of a phase III anifrolumab trial, Vital et al. further reported that baseline IFN gene set expression distinguishes molecular subgroups and is associated with treatment response [5], providing important insights into disease heterogeneity. Based on these findings, differential expression signatures have been matched to drug-induced transcriptional profiles from CMap and CLUE/L1000 to prioritize candidate compounds with potential to reverse disease-associated perturbations [6]. For example, Khunsriraksakul et al. studied SLE by examining genes associated with disease risk. They used CMap to rank drugs and pathways that might fix these genetic problems [7]. However, most of these studies focus on changes in gene expression levels. They do not pay enough attention to the systematic contributions at the post-transcriptional regulatory level. Moreover, although IFN modules account for a substantial fraction of heterogeneity, they do not fully capture the diversity of immune states in SLE. Therefore, identifying new disease subtypes, distinct from the usual IFN scoring systems, is important for improving accurate diagnosis in SLE and for better tailoring treatments.

Alternative polyadenylation (APA) is a key step in mRNA metabolism. APA generates mRNA isoforms with varying lengths of the 3′ untranslated region (3′UTR) by selecting different polyadenylation sites (PAS) [8]. APA is primarily categorized into two types: 3′UTR-APA, which adjusts 3′UTR length without altering coding sequences, and intronic-APA, which can produce truncated proteins [9]. Our study specifically focused on 3′UTR-APA. As a result, these changes affect mRNA stability, translation efficiency, and mRNA localization within cells [10,11]. APA is coordinated by a large set of APA regulators [12]. Growing evidence shows that APA regulators modulate PAS choice, remodel 3′UTR architecture, and thereby affect post-transcriptional gene regulation, with broad relevance to disease initiation and progression [13] [14]. Over the past decades, APA has gradually been recognized as one of the key regulatory mechanisms governing disease onset and progression. [[15], [16], [17]]. APA can alter the 3′UTR architecture and the regulatory environment, influencing critical processes such as immune receptor signaling, cytokine networks, antigen presentation, and cell fate determinations [[18], [19], [20]], and the regulatory role of APA has been repeatedly observed in cancer, infection, neurological disorders, and other inflammatory conditions [[20], [21], [22], [23]].In contrast, research on APA in SLE remains largely confined to studies of individual genes. For example, distinct APA events in GIMAP5 generate isoforms with different 3′UTR lengths, which can influence mRNA stability and protein abundance; this change has been implicated in disrupting T-cell homeostasis—particularly the balance between survival and apoptosis of regulatory T cells—thereby increasing SLE risk [24]. In addition, polymorphisms within the 3′UTR of the interferon regulatory factor IRF5 (e.g., rs10954213) may change the length and stability of the 3′UTR and are associated with SLE susceptibility [25,26]. These observations largely reflect single-gene examples, andthe immune-related APA landscape in SLE, and its potential clinical implications, has not been systematically elucidated.

Given this background, we used RNA-seq data from multiple clinical SLE cohorts to systematically characterize global APA events in SLE and identify APA events closely linked to immune pathways and states. We systematically identified immune-related APA events (ImmAPA) that may affect how SLE patients respond to therapy. We also found the key regulators associated with these APA changes in SLE. Next, we combined these ImmAPA events into a module and built an ImmAPA score to measure immune-related APA activity. It provides a post-transcriptional perspective for SLE stratification and may help generate hypotheses for treatment-related heterogeneity. Overall, our study adds a post-transcriptional regulatory dimension to SLE molecular subtyping and offers an interpretable framework for precision stratification and individualized therapeutic studies.

## 2 Materials and methods

### 2.1 Data resources

We obtained bulk RNA-seq data from the NCBI Gene Expression Omnibus (GEO). We used five SLE datasets: GSE72509 (whole blood; 99 SLE, 18 healthy controls (HC)), GSE110685 (whole blood; 62 SLE, 58 HC), GSE122459 (PBMCs; 20 SLE, 6 HC), GSE163073 (PBMCs; 20 SLE, 7 HC), and GSE175913 (sorted B-cell subsets; 24 SLE, 15 HC). We downloaded sequencing data and sample information files for each dataset. All analyses were conducted using available de-identified data, so we did not require additional ethical approval.

### 2.2 RNA-seq preprocessing, alignment, and gene expression quantification

Raw sequencing data (FASTQ format) went through quality control, filtering, alignment, and expression analysis. First, we used FastQC (v0.12.1) to check the quality of reads for each sample and MultiQC (v1.18) [27] combined FastQC. Generated a quality control report. The quality control metrics included per-base sequence quality, per-sequence GC content, sequence length distribution, adapter contamination, duplication levels, and overrepresented sequences. Next, Trimmomatic (v0.39) [28] was used to clean reads to produce FASTQ files for alignment. We downloaded the human reference genome GRCh38 and its annotation file from Ensembl (release 110) [29]. The reference genome index was built using STAR (v2.7.11a) [30]. Cleaned short reads were mapped to the GRCh38 reference genome using STAR. STAR output sorted BAM files and FASTQ files with unmapped reads for quality control or further analysis for each sample. After alignment, featureCounts (v2.0.6) [31] quantified gene-level read counts from BAM files. This step produced the raw gene expression count matrix. The counts were converted to TPM (or log2(TPM+1)) to enable expression analysis. TPM values were used to normalize for sequencing depth and gene length, and log2(TPM+1)-transformed expression values were used for downstream correlation analysis, ssGSEA, and differential expression analysis. Genes with all-zero TPM values or without valid expression values within a cohort were removed before downstream analyses. No direct cross-cohort expression matrix was generated for the main analyses.

### 2.3 APA event quantification, PDUI calculation and differentiation APA event identification

BedGraph and WIG files generated with BEDTools v2.30.0 [32] were used as inputs for DaPars2 v2.1 to detect APA events and quantify differential 3′UTR usage [19,33]. Briefly, sorted BAM files were converted into genome-wide coverage files using BEDTools, and the total number of mapped reads for each sample was recorded and included, together with the corresponding WIG file path in the DaPars2 configuration file. APA dynamics were summarized by the percentage of distal poly(A) site usage index (PDUI) output by DaPars2, which reflects the relative usage of distal poly(A) sites; higher PDUI values indicate increased distal site usage and a tendency toward 3′UTR lengthening. Differential APA events were therefore defined using FDR <0.05 and |ΔPDUI| > 0.1. Within each cohort, PDUI values were compared between SLE and healthy controls (HC) for each APA event. The Wilcoxon rank-sum test was used to assess the direction of effect and statistical significance (FDR<0.05). Events were classified as lengthening-type if PDUI(SLE) > PDUI(HC) with ΔPDUI >0.1. As shortening-type if PDUI(SLE) < PDUI(HC) with ΔPDUI >0.1 [34]. APA events that reach a significance threshold and are in the direction of at least two independent queues are defined as differential APA events. For cross-cohort integration, only events showing a consistent direction in at least two independent cohorts were retained as reproducible differential APA events.

### 2.4 APA regulators screening

We compiled a set of APA regulators from the literature [12]. Within each cohort, we calculate the correlation between the APA regulator expression and PDUI for each APA event using linear regression in both SLE and HC samples. For each factor, we calculated the percentage of substantially associated events (%) by dividing the number of APA events that exhibited a significant association post-FDR correction by the total number of events evaluated. We then compared the global shift in regulators–event association patterns between disease states (SLE vs HC) to characterize overall changes in regulatory coupling. We ranked the key twenty APA regulators based on (i) the strength of the regulators-event links and (ii) how consistent they were across cohorts.

### 2.5 Mendelian randomization analysis

To evaluate whether genetically predicted expression of candidate APA regulators has a causal impact on SLE susceptibility, we performed two-sample Mendelian randomization (MR) analyses. Genetic instruments for the exposure were obtained from GTEx [35] tissue-specific cis-eQTL summary statistics. MR analyses were conducted using the TwoSampleMR package (v0.6.2) [36] in R. Instrumental SNPs were chosen at P < 1 × 10^−5^ for their connection to target gene expression. They were then grouped based on linkage disequilibrium (LD) to retain variants that were mostly independent (r^2^ < 0.01 within a ±500 kb window). Effect alleles were aligned by harmonizing exposure and outcome datasets,and SNPs that could not be aligned or were ambiguous were excluded. Outcome data were SLE GWAS summary statistics (EFO/MONDO trait ID: MONDO_0007915). Primary causal estimates were obtained using the inverse-variance weighted (IVW) method, with effect estimates and P values reported.

### 2.6 ImmPathAPAScore

The ImmPathAPAScore framework was inspired by Gaoyang Wang et al. [37]. Four GEO RNA-seq cohorts (GSE72509, GSE110685, GSE122459, and GSE163073) were used, and ImmPort immune pathway gene sets were obtained from the ImmPort resource (https://immport.org/shared/genelists). The ImmPort collection contained 153 immune-related pathways. For each cohort and each APA event, we quantified the coupling between PDUI variation and transcriptome-wide expression patterns and projected this signal onto immune pathways as follows.

For a given APA event e, we computed the Spearman correlation between its PDUI values and the expression of each gene g, across samples, yielding a correlation coefficient ${\text{Corr}}_{e,g}$ and an associated P value $P_{e,g}$. We then defined a correlation score:

$${CS}_{e,g}=-\log_{10}\left(P_{e,g}\right)\times \text{sign}\left({Corr}_{e,g}\right)$$

Genes were ranked by $CS_{e,g}$, and gene set ssGSEA enrichment analysis was performed against ImmPort immune pathway gene sets to obtain enrichment statistics for each event–pathway pair, including enrichment direction (based on ES) and a nominal enrichment p-value (Ρ). The ssGSEA analysis was performed using the GSVA R package (v1.50.0) [38,39]. The event–pathway signal was then transformed into an ImmPathAPAScore:

$$\begin{array}{c} ImmPathAPAScore=1-2Ρ,\text{if}\;\text{ES}>0\;\\ \;2Ρ-1,if\;ES<0 \end{array}$$

To derive the R1 signal used for downstream filtering and prioritization, we used ImmPathAPAScore as a directional confidence filter rather than as the final enrichment magnitude. ImmPathAPAScore integrates the enrichment direction and the nominal P-value of each APA event–pathway pair, with values closer to +1 or −1 indicating stronger directional confidence. We then applied a binary thresholding function I(x) to determine whether the corresponding enrichment signal should be retained:

$$\begin{array}{c} R1=I\left(ImmPathAPAScore\right)*NES\text{,}\\ whereI\left(x\right)=\\ \;if\;\left|ImmPathAPAScore\right|<0.995,0\;\\ \;if\;\left|ImmPathAPAScore\right|\geq 0.995,1 \end{array}$$

Thus, R1 represents a thresholded NES signal. Specifically, R1 = 0 indicates that the APA event–pathway association did not pass the high-confidence ImmPathAPAScore threshold, whereas a non-zero R1 value indicates that the association was retained with its original NES direction and magnitude.

Pathway filtering. For each immune pathway, we calculated the proportion of events with R1=0 (the R1=0 ratio). Pathways with weak and unstable APA coupling were removed using a threshold of R1=0 ratio ≥0.95, and the remaining pathways were retained for subsequent ImmAPA identification. This pathway filtering step was performed to reduce the influence of immune pathways with limited or unstable APA coupling.

ImmAPA definition. Within the filtered pathway set, APA events were further required to (i) be reproducibly detected in at least two independent cohorts and (ii) show enrichment in at least 20 immune pathways. Events meeting both criteria were defined as ImmAPA. The requirement of recurrence in at least two cohorts was used to reduce cohort-specific signals, whereas the pathway-count requirement was used to prioritize APA events broadly coupled with multiple retained immune pathways.

### 2.7 Unsupervised hierarchical clustering based on ImmAPA with ImmAPA score, pathway score, and IFN score construction

We calculated ImmAPA scores, pathway scores, and IFN scores using single-sample gene set enrichment analysis(ssGSEA) [39] with the ImmAPA , immune pathway, and IFN gene sets, respectively. ssGSEA was performed on log2(TPM+1)-transformed expression matrices for each cohort, and the resulting scores were used for sample-level downstream analyses. ImmAPA grouping was performed through unsupervised clustering based on ImmAPA scores. Clustering analysis was performed independently for each dataset, using the k-means algorithm to partition samples into clusters. Multiple candidate cluster sizes were evaluated, and the contour width of each cluster was calculated to assess clustering separation. The cluster number was selected based on clustering separation metrics, including silhouette width, and the same clustering strategy was applied across cohorts.

### 2.8 Connectivity Map–based drug prioritization using subtype-specific transcriptional signatures

Healthy controls (HC) were used as the reference group. Separate tests for differential expression were performed between ImmAPA-high and HC, and between ImmAPA-low and HC. Differential expression analysis was conducted using the limma R package (v3.50.3, Bioconductor) [40]. LogFC, P values, and FDR were reported for each gene. The same cutoffs were applied across all comparisons. Differentially expressed genes (DEGs) were defined as those with ∣logFC∣>0.5 and FDR <0.1. We split DEGs into upregulated and downregulated sets, which were then used to construct subtype signatures. Each signature was submitted to CMap for perturbation matching. Candidate compounds and their normalized connectivity scores were obtained from CMap. Lower negative scores were interpreted as stronger reversal signals, which were used to prioritize candidate compounds for further investigation.

### 2.9 Statistical analysis and visualization

Two-sided Wilcoxon rank-sum tests were used to compare continuous variables between two groups, and Spearman's rank correlation was used. The FDR procedure was used to address multiple testing. Unless otherwise specified, all statistical tests were two-sided. Figures were generated primarily in R (e.g., ggplot2, ComplexHeatmap) and assembled for publication using Adobe Illustrator and/or BioRender. Statistical significance was annotated as follows: *P < 0.05, **P < 0.01, ***P < 0.001, ****P < 0.0001 [41]. All statistical analyses were performed in R (v4.1.3) and Python (v3.11.4). The main software and packages included FastQC (v0.12.1), MultiQC (v1.18), Trimmomatic (v0.39), STAR (v2.7.11a), featureCounts (v2.0.6), BEDTools (v2.30.0), DaPars2 (v2.1), limma (v3.50.3), TwoSampleMR (v0.6.2), and coloc (v5.2.3). APA quantification, differential APA analysis, ImmPathAPAScore calculation, ssGSEA scoring, and clustering were performed independently within each cohort unless otherwise stated. Cross-cohort integration was performed only at the level of reproducible events or summary statistics.

## 3 Result

### 3.1 Systematic alteration of alternative polyadenylation in SLE

**Fig. 1:**
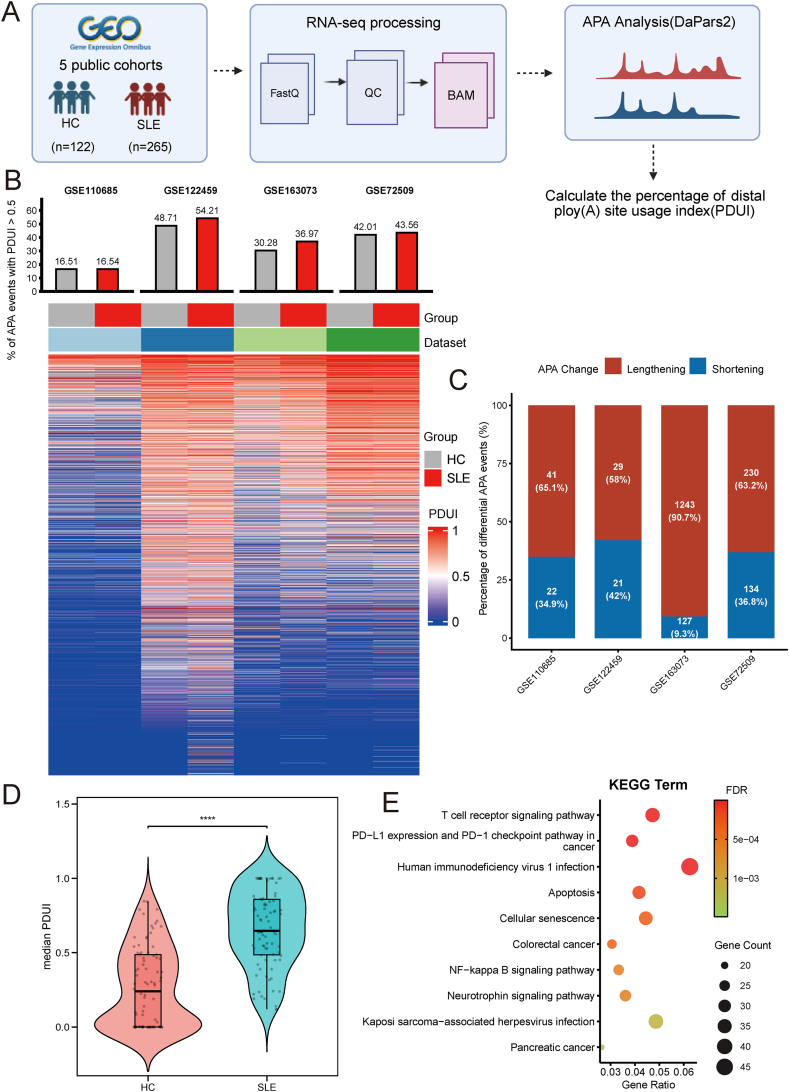
Global 3′UTR lengthening characterizes the APA landscape in SLE (A) Schematic diagram of the APA event quantification workflow: Bulk RNA-seq data from multiple cohorts of SLE and HC were obtained from GEO. Following quality control and alignment, APA quantification was performed using DaPars2, and the PDUI was calculated. (B) PDUI heatmap of all APA events at the sample level. The columns are arranged by samples, and the rows are arranged by PDUI of each APA event across all samples. The top annotation bar labels indicate group (HC/SLE) and dataset, with SLE shown in red and HC shown in gray. In the heatmap, red indicates transcripts with PDUI >0.5, while blue indicates transcripts with PDUI <0.5. (C) Directional composition of differential APA events in each independent cohort. Events are classified by the direction of PDUI change within each cohort. Numbers within bars indicate event counts and proportions. (D) Comparison of PDUI distributions for concordant differential APA events across cohorts. Concordant events were defined as differential APA events detected in at least two independent cohorts with consistent directionality. Each point represents one event; the y-axis denotes the cohort-specific median PDUI. Differences between groups were evaluated using a two-sided Wilcoxon rank-sum test. Significance: *P < 0.05; **P < 0.01; ***P < 0.001; ****P < 0.0001. (E) KEGG pathway enrichment analysis of genes corresponding to cross-cohort concordant lengthening differential APA events. Bubble size represents the number of enriched genes (gene count), and color indicates the multiple-testing–adjusted significance level (FDR).

To systematically profile the global APA landscape in SLE, we compared PAS selection between SLE patients and healthy controls (HC) across multiple cohorts (Table S1) using the PDUI, as outlined in Fig. 1A. Results revealed significantly higher overall PDUI values in SLE patients compared to HC (Fig. 1B), indicating increased use of distal PAS and global 3′UTR lengthening. In both whole-blood and PBMC cohorts, 3′UTR lengthening constituted a high proportion of differential APA events, ranging from 58.0% to 90.7% across datasets (Fig. 1C). Conversely, the B-cell dataset (GSE175913) exhibited a greater proportion of 3′UTR-shortening events compared to lengthening events (Supplementary Fig. 1A). Given its cell-type-specific sample source and distinct APA directional pattern, this B-cell dataset was excluded from subsequent cross-cohort integrative analyses to maintain comparability across cohorts. After intersecting differential APA events detected in at least two datasets, the shared events (n = 86) also showed significantly higher PDUI values in SLE than in HC (Fig. 1D). These findings suggest that APA alterations in SLE are not limited to isolated changes in individual events, but instead a recurrent bulk transcriptome-level APA pattern observed across multiple SLE cohorts, characterized by 3′UTR lengthening. This supports the potential of the PDUI index to distinguish SLE patients from HC.

To explore the potential functional relevance of these APA lengthening events, we conducted pathway enrichment analysis on genes consistently associated with lengthening APA across multiple datasets (Fig. 1E; Supplementary Fig. 1B and C). These genes were significantly enriched in immune signaling pathways closely implicated in SLE pathogenesis, including T cell receptor signaling, the PD-L1 expression and PD-1 checkpoint pathway in cancer, NF-κB signaling, and immune system-related pathways [[42], [43], [44], [45]]. These pathways are associated with breakdown of immune tolerance, aberrant interferon signaling, dysregulated T and B-cell activation, excessive cytokine production, and the maintenance of chronic inflammation in SLE. These suggest that 3′UTR lengthening is associated with SLE-related immune processes and may reflect an additional layer of post-transcriptional regulation in SLE.

### 3.2 Key APA regulators drive 3′UTR lengthening APA events in SLE

**Fig. 2:**
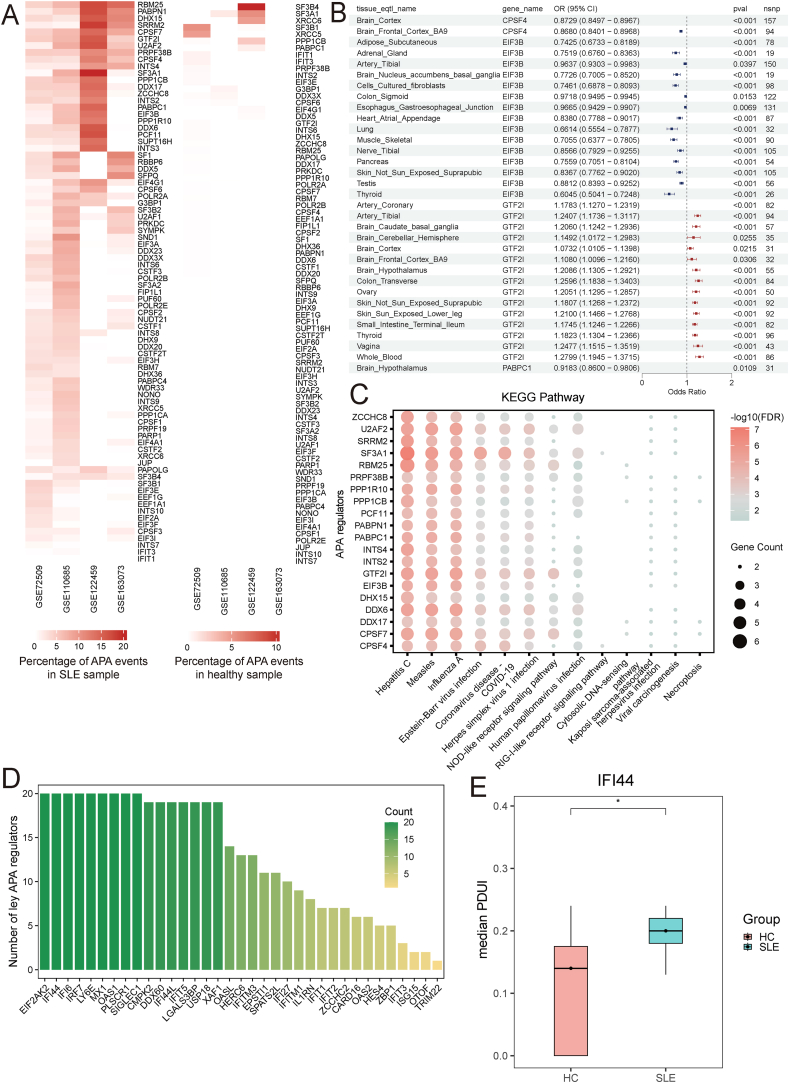
APA regulators are associated with APA remodeling in SLE. (A) Across four independent cohorts (GSE72509, GSE110685, GSE122459, and GSE163073), associations between the expression of APA regulators and APA events (PDUI) were evaluated separately within SLE and HC samples. The percentage of significantly associated APA events for each regulator was summarized. The left heatmap corresponds to SLE and the right heatmap to HC; colors denote the percentage of significant regulator–event associations. (B)Using tissue-specific eQTL reference data, we performed two-sample MR to evaluate the association between genetically predicted expression of candidate core APA regulators and SLE risk. The forest plot reports MR-derived causal effect estimates as odds ratios (ORs) with 95% confidence intervals (CIs), along with *P* values and the number of instrumental SNPs (nsnp); estimates were obtained using the inverse-variance weighted (IVW) method. (C) KEGG pathway enrichment analysis of gene sets associated with twenty key APA. Bubble size indicates the number of enriched genes (gene count), and color represents multiple-testing–adjusted significance [−log10(FDR)], summarizing immune and infection related functional modules potentially linked to different regulators. (D) Summary of representative genes potentially associated with APA regulators, ranked by the number of Top20 APA regulators associated with each gene. The y-axis indicates the number of associated key regulators per gene; the color gradient likewise reflects the counts, highlighting genes potentially associated with multiple regulators. (E) PDUI distribution of the APA event corresponding to the representative gene *IFI44* in HC versus SLE. The y-axis indicates median PDUI. Differences between groups were evaluated using a two-sided Wilcoxon rank-sum test. Significance: *P < 0.05; **P < 0.01; ***P < 0.001; ****P < 0.0001.

To identify key regulators associated with global APA changes in SLE, we systematically analyzed association patterns between 83 reported APA regulators [12] and genome-wide APA events. Using a linear regression framework [20], we compared the correlation structure between APA regulators and APA events in SLE and HC. In all cohorts, the strength of associations was higher in SLE, and the overall association architecture differed from HC (Fig. 2A). Positive correlation events were more common than negative ones in SLE (Supplementary Fig. 2A), suggesting that many APA regulators in SLE are associated with increased use of distal PAS. This finding is consistent with the previously observed overall increase in PDUI and global 3′UTR lengthening in SLE, indicating that APA lengthening in SLE may be related to altered APA regulator–event association patterns rather than random.

Using the ranking from the linear model, we identified twenty key APA regulators with the widest regulatory role for further investigation. MR analysis showed that genetically predicted expression levels of several variables, including *CPSF4*, *EIF3B*, *GTF2I*, and *PABPC1*, were associated with the SLE risk across multiple tissues (Fig. 2B). These findings indicate that APA regulators may play a significant role in post-transcriptional regulation, and their genetic variants may be linked to SLE susceptibility. This provides genetic evidence supporting their potential relevance to the biology of SLE.

To further characterize the functional roles of these APA regulators, we conducted KEGG pathway enrichment analysis for the genes corresponding to APA events associated with each regulator and synthesized enriched pathways across regulatory (Fig. 2C). The findings showed that gene sets associated with different APA regulators converge on similar KEGG pathways, primarily dominated by antiviral innate immune programs, including RIG-I–like receptor signaling, cytosolic DNA sensing, NOD-like receptor/inflammasome signaling, and several viral infection pathways. These pathways share core modules highly relevant to SLE, including type I interferon signaling, NF-κB–driven inflammation, antigen processing and presentation, and cellular stress responses. Therefore, despite the varied origins of APA regulators, their downstream associated genes exhibit functional convergence, suggesting that APA regulation in SLE may involve a coordinated process among multiple related immune pathways.

Next, we integrated APA events associated with twenty key APA regulators with differentially expressed genes (DEGs) in SLE. The overlapping genes showed an overall upregulation trend in SLE (Supplementary Fig. 2B). IFN-responsive genes such as *EIF2AK2* and *IFI44* were associated with multiple APA regulators and simultaneously displayed marked upregulation and pronounced 3′UTR lengthening in SLE (Fig. 2D and E). De Cevins et al. also reported that variants in *ZCCHC8* and *EIF2AK3* can induce core amino acid changes and are associated with the autoinflammatory disease SAVI [46]. These results indicate that 3′UTR lengthening in SLE preferentially affects a subset of immune effector genes involved in interferon and inflammatory responses, rather than occurring randomly.

To evaluate the roles of these key APA regulators, we consulted the existing literature on these 20 APA regulators. Half (n = 10) of these regulators had been reported in studies of autoimmune diseases such as SLE or lupus nephritis (Table 1), supporting the relevance of APA-related regulation in autoimmunity. The remaining regulators were previously associated only with tumors or other diseases (Table S2). This study introduces these non-classical autoimmune regulators into the regulatory network of SLE, suggesting that shared mechanisms of APA dysregulation may exist across different pathological states and may broaden our understanding of APA in the context of SLE. To further assess whether these APA regulators form functional cooperation at the protein level, we constructed a protein–protein interaction (PPI) network using the STRING database [58]. As shown in Supplementary Figure2 C, many APA regulators displayed dense interconnections, and their high connectivity within the network suggests that APA lengthening in SLE may involve interactions among multiple APA proteins. Functional enrichment analysis based on STRING annotations (Supplementary Fig. 2D), and showed that enriched terms were primarily related to PAS recognition and regulation of cleavage efficiency. These results suggest that many APA regulators may cooperate in distal PAS selection and may be associated with 3′UTR lengthening in SLE.

**Table 1:** Function of the twenty key APA regulators.

APA regulators	Functions	Reference
RBM25	Associated with certain related autoimmune diseases (such as RA); interacts with DDX11, a key gene in SLE, through protein networks.	*Zhang* et al., 2024 [47]; *Saeed* et al. ,2021 [48]
DHX15	As an aberrantly regulated splicing factor, it shows differential expression in SLE and represents a potential therapeutic target for SLE.	*Xu* et al., 2025 [49]
SRRM2	Identified as SLE targets in primary B cells and plasma blasts from SLE patients	*Pullabhatla* et al., 2018 [50]; *Rivellese* et al., 2021 [51]
GTF2I	GTF2I polymorphism may serve as a useful predictor of renal dysfunction in patients with SLE.	*Meng,*et al., 2019 [52]
PPP1CB	The Ras-MAPK pathway is upregulated in lupus nephritis, contributing to disease progression.	*Zhang* et al., 2025 [53]
DDX17	In SLE, its expression in immune cells negatively correlates with the X/autosomal (X/A) transcript ratio; inhibiting DDX17-mediated NLRC4 inflammasome activation reduces interleukin-18 (IL-18) release in peripheral blood mononuclear cells from SLE patients.	*Soares* et al., 2025 [54]; *Wang* et al., 2021 [55]
ZCCHC8	SAVI Syndrome with Induced Lupus-Like Phenotype	*de Cevins* et al., 2023 [46]
PABPC1	In monocytes, it is a predictor of SLE.	*Soares* et al., 2025 [54]
EIF3B	Autoantibodies against a novel autoantigen, the eIF3 complex, were detected in idiopathic inflammatory myositis, but they were not directly associated with SLE.	*Betteridge* et al., 2020 [56]
DDX6	It is a genetic risk locus for SLE (DDX6-CXCR5); it affects immune cell regulatory networks; its overexpression correlates with IFN responses.	*Wiley* et al., 2023 [57]

In summary, APA remodeling in SLE is associated with several key APA regulators. These core regulators generally promote distal PAS usage and target genes involved in antiviral responses and interferon signaling. By altering 3′UTR usage in immune effector genes, which may affect RBP binding and immune receptor signaling, this APA program could con tribute to abnormal immune activation in SLE.

### 3.3 Integrative immune pathway analysis identifies ImmAPA

**Fig. 3:**
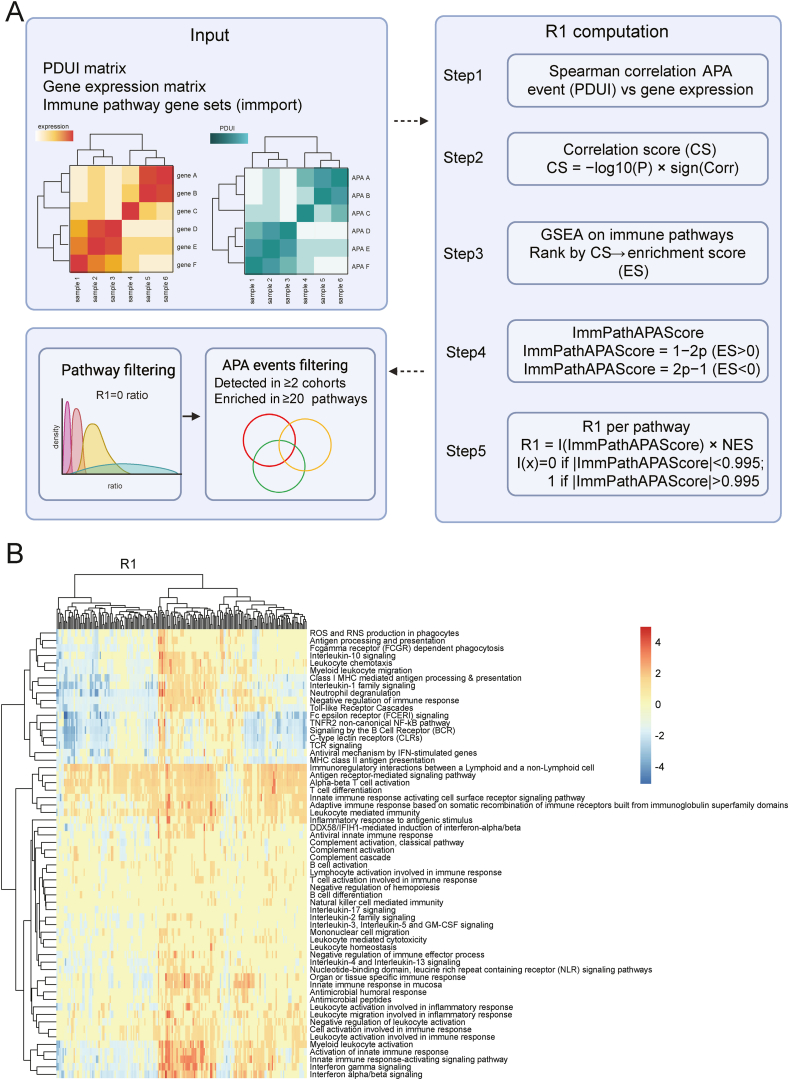
Workflow for defining mmAPA based on ImmPathAPAScore. (A) Schematic overview of the ImmAPA identification pipeline. Inputs include the APA-event PDUI matrix, the gene-expression matrix, and immune pathway gene sets curated from ImmPort. ImmPathAPAScore was calculated to summarize the direction and statistical confidence of each APA event–immune pathway association, followed by R1-based pathway filtering and cross-cohort ImmAPA event selection. (B) The heatmap displays the R1 between immune pathways and APA events. Colors represent the strength and direction of R1 (red indicates positive correlation, blue indicates negative correlation).

Although our analysis shows widespread APA-associated 3′UTR prolongation in SLE, it could not distinguish which events are closely linked to the SLE immunopathological regulatory network and which represent accompanying changes. Since APA can modulate immune responses by altering transcript stability, translational efficiency, and miRNA binding [[59], [60], [61]], we developed a pathway-level framework, ImmPathAPAScore, tailored to our datasets to systematically quantify the coupling between APA dynamics and immune pathway activity (see 2.6; workflow in Fig. 3A). By integrating PDUI shifts in APA events with immune pathway activity scores, this approach provides a functional view of the potential contribution of APA to immune regulation in SLE.

We utilized four public SLE datasets to obtain PDUI values for APA events and their corresponding gene expression matrices. By integrating immune pathway gene sets from the ImmPort database, we derived R1 values for each APA event across every pathway. Next, we filtered out pathways where the proportion of R1 = 0 events exceeded 0.95, retaining immune pathways with high APA enrichment. We then selected APA events that were reproducibly detected in at least two independent cohorts and enriched in at least 20 immune pathways. Events satisfying these criteria were defined as ImmAPA. This approach identified 202 key ImmAPA events (Table S3), which served as the core event set for subsequent stratification and phenotype association analyses.

By integrating immune pathways and APA events, we observed differences in ImmPathAPAScores across distinct immune pathways (Fig. 3B). Some pathways showed consistent, concentrated enrichment across multiple APA events. These findings suggest that APA alterations are preferentially coupled with specific immune functional modules rather than uniformly affecting all immune processes. This insight advances our understanding of APA-related immune dysregulation in SLE.

### 3.4 ImmAPA score defines a complementary dimension of immune activation and stratifies SLE patients

**Fig. 4:**
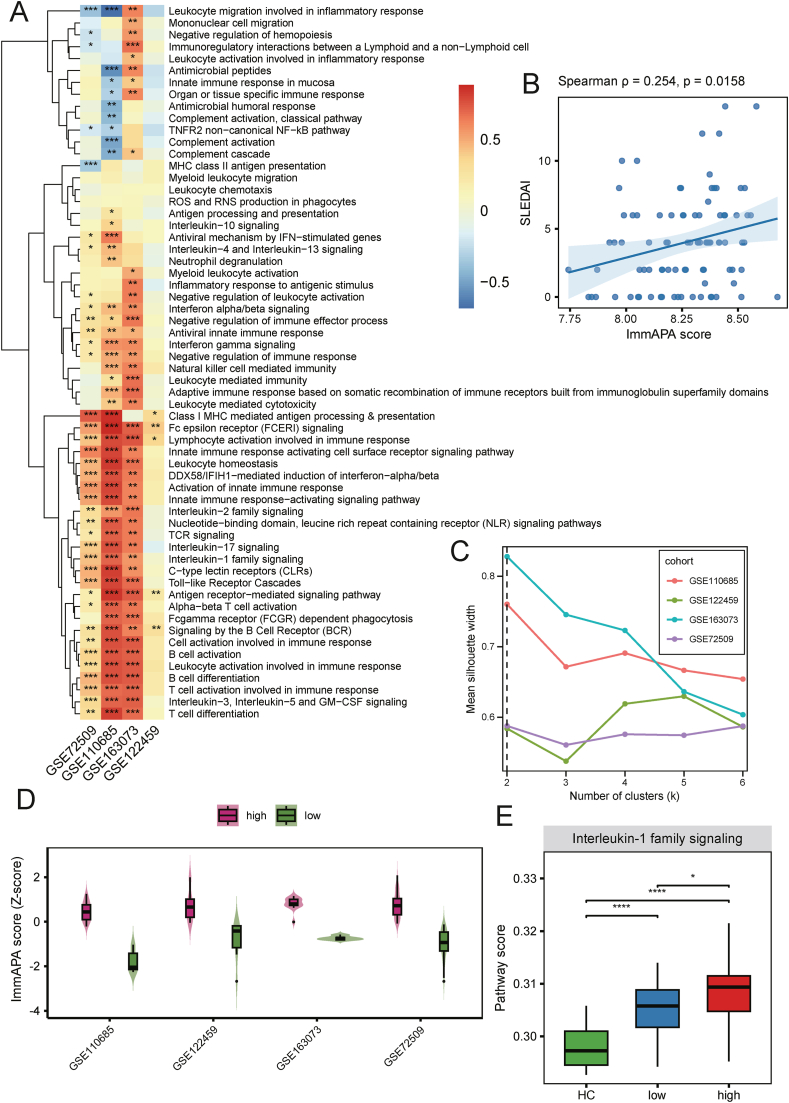
ImmAPA score is associated with immune pathway activity and stratifies SLE patients. (A) Correlation analysis of ImmAPA score and pathway score constructed based on ImmAPA events across four independent SLE cohorts. Each row represents one retained immune pathway, and each column represents one cohort. Spearman correlation coefficients between ImmAPA score and pathway score, with red indicating positive correlations and blue indicating negative correlations. (B) Spearman correlation analysis between SLEDAI and ImmAPA score (ρ = 0.254, p = 0.0158). Each plots represents one SLE patient. The fitted line and shaded area indicate the linear trend and confidence interval. The correlation coefficient and P value are shown above the plot. (C) Line plots of mean silhouette width versus number of clusters (k) across different datasets to assess clustering quality. The silhouette analysis was used to assess clustering structure and support the selection of two ImmAPA-based patient groups. (D) Distribution of scaled ImmAPA scores in ImmAPA-high and ImmAPA-low groups across four independent cohorts. ImmAPA scores were standardized within each cohort and are shown as Z-scores. The violin and box plots show the distribution of ImmAPA activity in the two groups, supporting the reproducibility of ImmAPA-high and ImmAPA-low stratification across cohorts. (E) Pathway score analysis of Interleukin−1 family signaling across HC, ImmAPA-low, and ImmAPA-high groups. Group comparisons were performed using the two-tailed Wilcoxon rank-sum test. Significance annotation rules: *P < 0.05, **P < 0.01, ***P < 0.001.

We performed MR analyses for the 156 genes corresponding to the 202 ImmAPA events and found that 23.7% of these genes showed genetic evidence consistent with a potential effect of gene expression on SLE risk (Supplementary Fig. 3A). We next investigated the overall relevance of ImmAPA. Treating ImmAPA events as a functional module, we calculated an ImmAPA activity score (ImmAPA score). We also computed pathway activity scores (Pathway score) for the 60 retained immune-related pathways, enabling a multi-dimensional assessment of the association between APA regulation and immune pathway activity. Across four independent SLE cohorts, correlation analyses showed that the ImmAPA score was consistently and positively associated with the activity of most immune pathways (Fig. 4A). Pathways tightly linked to SLE pathogenesis, including Activation of innate immune response, Fc epsilon receptor (FCERI) signaling, and Interleukin-2 family signaling, exhibited directionally consistent and significant correlations across multiple cohorts. These indicate that the ImmAPA score is associated with immune pathway activation levels.

Next, we looked at how ImmAPA-related activity and clinical disease activity, as measured by the SLE Disease Activity Index (SLEDAI). InFig. 4B, the ImmAPA score showed a moderate but statistically significant positive correlation with SLEDAI (Spearman ρ = 0.254, p = 0.0158), suggesting that higher immune-related APA activity is associated with increased SLEDAI. In contrast, when applying the same strategy to compute an IFN gene set score (IFN score), its correlation with SLEDAI was weaker and not significant (ρ = 0.116, p = 0.278)(Supplementary Fig. 3D). The ImmAPA score also had only a low correlation with the IFN score (Supplementary Fig. 3E). It suggests that the ImmAPA score may capture information not fully represented by the IFN score. To further evaluate whether the ImmAPA score provides additional information beyond the IFN score, we performed a nested linear regression analysis. We first fitted a model using the IFN score alone, then added the ImmAPA score. The IFN-only model showed limited explanatory performance for SLEDAI, whereas the model including both IFN score and ImmAPA score showed improved model fit (Supplementary Fig. 3F). These results suggest that the ImmAPA score provides complementary SLEDAI-related information beyond the traditional IFN score, although the overall explanatory power of the model remains limited.

Analysis of the sample distribution revealed a clear bimodal pattern in ImmAPA scores across datasets (Supplementary Fig. 3B and 3C). This distribution suggests heterogeneity in ImmAPA regulation among SLE patients. Based on this, we classified patients into ImmAPA-high and ImmAPA-low groups, a distinction that remained stable across four independent cohorts (Fig. 4C). This suggests that the ImmAPA classification captures reproducible molecular heterogeneity within SLE.

To evaluate biological relevance, we compared immune pathway characteristics among HC, ImmAPA-low, and ImmAPA-high groups. The ImmAPA classification distinguished differences in immune activation across these groups. Activation levels varied between ImmAPA-high and ImmAPA-low groups depending on the pathway (Supplementary Fig. 4A), highlighting the value of classification. Among these ImmAPA-associated immune pathways, Interleukin-1 family signaling showed a clear difference among HC, ImmAPA-low, and ImmAPA-high groups and was selected as a representative inflammatory pathway for visualization. Interleukin-1 family signaling, reflected by serum IL-1 levels, which have been reported to be elevated in SLE and positively associated with disease activity [62], was significantly higher in the ImmAPA-high group (Fig. 4E). The ImmAPA score showed reproducible patterns across independent cohorts, distinguishing immunological activation states in patients with SLE, and was associated with SLEDAI. These findings suggest that ImmAPA stratification, derived from APA regulatory features, captures SLE immune heterogeneity from a post-transcriptional regulatory perspective and provides a foundation for downstream mechanistic investigations.

Our study supports that the ImmAPA score is a reproducible post-transcriptional activity score associated with immune activation and disease activity in SLE. This score provides complementary information to the conventional IFN score in evaluating the SLEDAI. As an independent indicator, its results are not entirely dependent on IFN levels and may help identify molecular heterogeneity that is not fully captured by conventional IFN measures. Importantly, ImmAPA grouping shows molecular differences among SLE patients, distinguishing between ImmAPA-high and ImmAPA-low subgroups. We therefore hypothesize that ImmAPA subgroups may exhibit different treatment-related transcriptional patterns, which were further explored in subsequent analyses.

### **3.5** **ImmAPA-defined subtypes** are associated with **transcriptomic remodeling and therapeutic response**

We used CIBERSORTx [63,64] to analyze inferred immune cell composition in the two patient groups. The results showed differences in the immune composition between the subtypes (Supplementary Fig. 5A). The ImmAPA-high group showed a significant increase in CD14^+^ monocytes proportion in multiple cohorts, which may partly contribute to the higher inflammatory pathway activity observed in this group. In contrast, memory B cell proportionwere lower in the ImmAPA-high group in some cohorts. However, these differences were not uniformly significant across all datasets, suggesting that immune cell composition may contribute to, but not fully explain, ImmAPA-associated immune pathway activity.

**Fig. 5:**
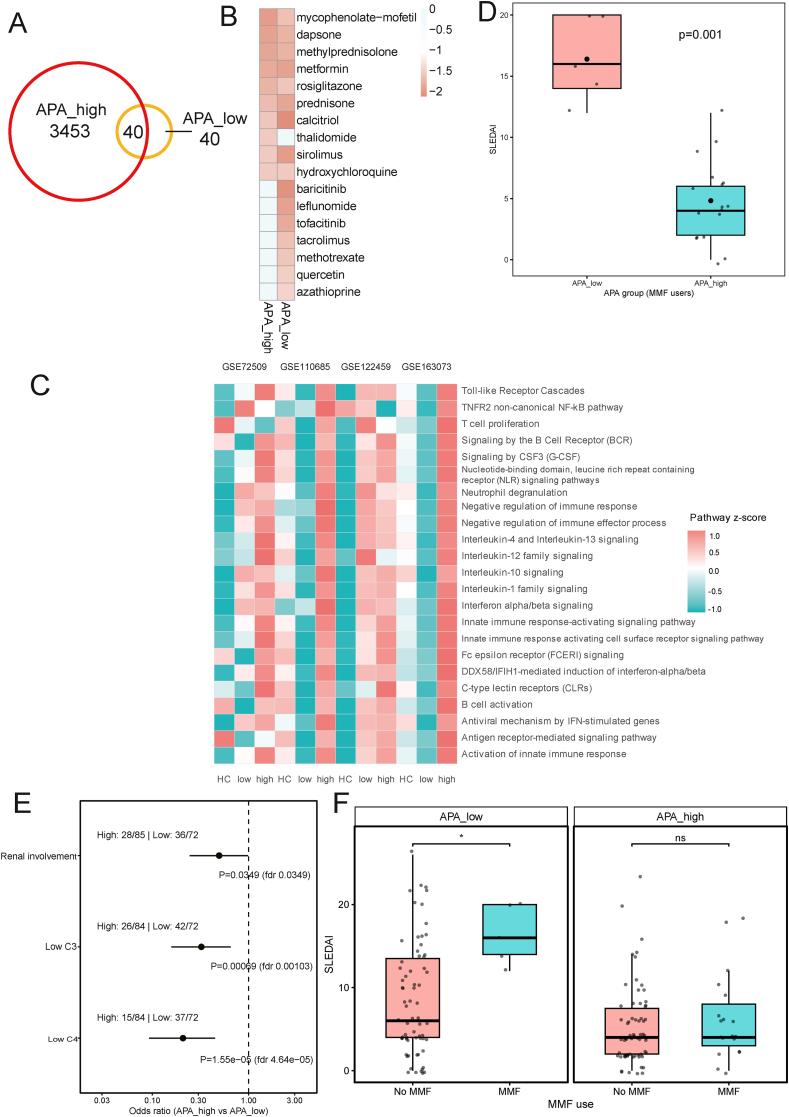
ImmAPA stratification reveals subtype-associated transcriptomic perturbation, drug-related transcriptional connectivity, and clinical features in MMF-treated patients. (A) Differential expression analyses were performed for ImmAPA-high (APA_high) and ImmAPA-low (APA_low) subgroups defined by APA score, using HC as the reference. A Venn diagram summarizes the size and overlap of subgroup-specific differentially expressed gene (DEG) sets. (B) CMap–based drug transcriptional connectivity analysis was conducted using the subgroup-specific transcriptional signatures. The heatmap shows the candidate drugs identified for APA_high and APA_low. The colors show the normalized connectivity score, with more negative values meaning a stronger predicted inverse connectivity with subgroup-associated transcriptional signatures. (C) Heatmap of immune-related pathway activity differences among HC, APA_low, and APA_high across four independent cohorts (GSE72509, GSE110685, GSE122459, and GSE163073), represented as pathway z-scores. Left-side links indicate associations between candidate compounds and their corresponding immune pathway sets. (D) In an external MMF GSE49454 follow-up cohort, follow-up SLEDAI was compared between APA_low and APA_high within MMF users; two-sided *P* values are shown. (E) Selected clinical features associated with APA_high relative to APA_low, including renal involvement, low C3, and low C4, are presented as odds ratios (ORs) with confidence intervals. Corresponding *P* values and FDR-adjusted significance are indicated. (F) Within APA_low and APA_high subgroups, the relationship between MMF use and follow-up SLEDAI was evaluated. Group comparisons were performed using two-sided nonparametric tests; significance is denoted by asterisks, and ns indicates not significant. Significance annotation rules: *P < 0.05, **P < 0.01, ***P < 0.001.

Since immune cell activation in SLE is associated with disease progression, we compared DEGs in both patient groups to HC. Our results show that these two groups possess distinct molecular characteristics. We observed a massive transcriptomic shift in the ImmAPA-high group, which involved 2493 DEGs (Fig. 5A). The ImmAPA-low group showed fewer gene changes, with only 80 DEGs. The overlap between the two subgroup signatures was limited to a small gene set (n = 40), indicating that ImmAPA-high is associated with broad immune transcriptional activation, whereas ImmAPA-low reflects low activation or a more localized perturbation phenotype (Fig. 5A and Supplementary Fig. 5B).

We conducted a focused literature review of commonly used medications for SLE. We compiled an SLE drug list and identified 17 drugs that were also available in the CLUE database (Table S4). We matched subgroup-specific DEGs to drug induced transcriptional signatures to explore potential transcriptional connectivity patterns. The results showed that all 17 drugs produced reproducible connectivity scores across the subgroup signatures. These drugs included immunosuppressive and anti-inflammatory agents such as mycophenolate-mofetil (MMF), methotrexate, azathioprine, hydroxychloroquine, and glucocorticoids.

Different drugs showed distinct connectivity scores for the ImmAPA-high and ImmAPA-low signatures (Fig. 5B), suggesting that ImmAPA-defined subgroups may exhibit distinct predicted transcriptional connectivity patterns to drug perturbation signatures. To interpret these findings, we integrated each drug's mechanism of action (MOA) (Supplementary Fig. 5C). The analysis showed that candidate MOAs were mainly concentrated in immune-relevant target classes, including dehydrogenase inhibitors, JAK inhibitors, and insulin sensitizers. We also mapped these drugs to the panel of 60 immune pathways. The mapping showed that several candidate drugs were associated with higher immune pathway activation in ImmAPA-high than in ImmAPA-low (Fig. 5C), suggesting potential subgroup-related differences in immune pathway context.

Afterward, we used an external treatment follow-up cohort (GSE49454, Table S5) to conduct an exploratory treatment-associated analysis. We divided patients into ImmAPA-high and ImmAPA-low groups based on baseline ImmAPA status, using MMF as an example. In MMF users, ImmAPA-high patients exhibited a lower post-treatment follow-up SLEDAI than ImmAPA-low patients (Fig. 5D), indicating that ImmAPA stratification may capture clinical heterogeneity within an MMF-treated subgroup. ImmAPA-high was associated with selected clinical features, including a lower prevalence of renal involvement and hypocomplementemia, as reflected by low C3 and low C4, compared with ImmAPA-low (Fig. 5E). These observations suggest an association between baseline ImmAPA status and selected clinical features in this external cohort.

Treatment allocation and dosing may be influenced by disease severity, as physicians tend to reduce medication for less severe cases. Consequently, SLEDAI may be higher among MMF users than non-users in the ImmAPA-low subgroup, whereas no significant difference was observed in the ImmAPA-high subgroup (Fig. 5F). This indicates that disease progression is controlled by medication in the ImmAPA-high group. Even so, we still observed a clear distinction between the ImmAPA-high and ImmAPA-low groups within MMF users. These findings suggest that our ImmAPA classification may help describe patient heterogeneity in treatment-related contexts, but prospective cohorts with standardized treatment protocols and functional validation are required.

## 4 Discussion

In this study, we analyzed patient data to investigate APA in SLE and developed the ImmAPA module and ImmAPA score. Unlike previous research that focused mainly on gene expression and IFN patterns, we identified recurrent APA alterations in SLE patients, resulting in two distinct groups: ImmAPA-high and ImmAPA-low. This approach offers a post-transcriptional perspective on the heterogeneity of SLE and complements traditional gene expression analyses.

In SLE, APA is characterized by altered polyadenylation site usage, with a predominant shift toward distal PAS usage and widespread 3′UTR lengthening. A longer 3′UTR may provide additional binding sites for miRNAs and RBPs [59,65], thereby increasing regulatory complexity [34,56] and influencing mRNA stability [8]. This trend was observed across multiple independent patient cohorts. These changes were also associated with specific biological functions. Therefore, these findings suggest that APA remodeling represents a recurrent bulk transcriptome-level pattern observed across multiple SLE cohorts.

We further explored potential regulators associated with these APA changes. Among the 83 reported APA factors, the twenty with the strongest regulatory effects in SLE were identified, forming a highly connected network. These findings indicate that increased distal PAS usage may be related to coordinated alterations of multiple APA regulators, rather than a single regulator. Similar mechanisms have been reported in other diseases. For example, during an influenza A virus (IAV) infection, the virus uses its NS1 protein to attack the host CPSF4 protein. This changes the 3′UTR length and controls the level of inflammation [22]. Our MR data further demonstrate that genetically predicted expression of some APA regulators was associated with SLE risk, supporting their potential relevance to SLE disease biology, although these results do not establish direct causality.

We identified distinct immune functional modules by using ImmPathAPAScore, termed ImmAPA. The data suggest that the ImmAPA selectively influences specific immune pathways, including innate immunity, the NF-κB inflammation chain, and antigen processing. This finding supports the prevailing view that SLE involves multiple immune pathways simultaneously [66]. More importantly, the ImmAPA score was associated with immune pathway activity and showed a positive correlation with SLEDAI. Its correlation with the traditional IFN score was weak, suggesting that the ImmAPA score may capture post-transcriptional information not fully represented by IFN-based measures. In addition, nested linear regression analysis showed that adding the ImmAPA score to the IFN score improved model fit for SLEDAI, although the overall explanatory power remained limited. Therefore, ImmAPA should be interpreted as a complementary post-transcriptional layer associated with disease activity, rather than a completely independent or clinically validated biomarker.

When we look at bulk tissue or peripheral blood transcriptomes, the APA events may be influenced by differences in immune cell composition. The data revealed distinct differences in the proportions of monocytes and B cells between the ImmAPA-high and ImmAPA-low groups. In a separate B cell dataset, the opposite trend in 3′UTR length was observed. These findings highlight that APA regulation is highly cell-type specific and that tissue-level changes result from both shifts in cell composition and altered processing within individual cell types.

For clinical application, we used the CMap [67,68] to identify potential drug candidates based on ImmAPA subgroup-associated transcriptional signatures. This analysis provides a hypothesis-generating approach for exploring drug-related transcriptional connectivity across different SLE patient subgroups. In patients treated with MMF [52,53], the initial ImmAPA score matched the extent of their recovery. This method of matching drug effects to gene changes yields only hypotheses. Variables such as drug type, dose, and timing can influence the efficacy of these projections in practical scenarios. Current evidence shows ImmAPA groups are associated with disease progression during treatment, but the predictive value for drug response remains unconfirmed. Future research should test the ImmAPA score in rigorous clinical trials to determine whether it is a reliable tool for patient grouping.

Several limitations should be acknowledged. While our analysis highlighted potential key aspects of ImmAPA events and regulators, experimental validation remains essential. Future investigations could confirm representative ImmAPA events through isoform-specific qPCR, 3′RACE, long-read sequencing, reporter assays, or APA regulator perturbation experiments in immune cells. As a post-transcriptional regulatory mechanism, APA is tissue-specific and varies among cell types. Our current framework relies on bulk RNA-seq data, which may be sensitive to variations in immune cell composition. Future improvements could include analyzing tissue- and cell type-specific APA events, such as granulocyte-related events, which are closely linked to SLE pathogenesis. Incorporating immune cell proportions as covariates, or adjusting ImmAPA score and pathway scores for major cell composition factors, may enhance accuracy. Stratified analyses based on sample source, such as whole blood and PBMC, along with single-cell or cell-type-specific APA profiling, will further clarify this issue. Heterogeneity among cohorts may also affect ImmAPA–pathway correlations. For example, GSE122459 is a small PBMC RNA-seq dataset with 20 SLE patients and six controls, and was generated using different sequencing platforms. Weaker ImmAPA–pathway correlations may be the result of differences in sample source, cell composition, batch effects, or incomplete public clinical annotation. Public treatment cohorts often lack complete clinical annotations and may be affected by concomitant medications and treatment indication confounding. Therefore, prospective cohorts with standardized treatment regimens, complete clinical annotations, and longitudinal follow-up are required to evaluate the clinical utility of ImmAPA stratification.

In conclusion, our study expands the understanding of post-transcriptional regulation in SLE. The ImmAPA framework enables patient stratification from the perspective of APA regulation, suggesting that SLE heterogeneity extends beyond gene expression to 3′-end processing and post-transcriptional regulation. These findings provide an interpretable framework for future mechanistic studies and hypothesis-generating precision stratification in SLE.

## Ethics statement

This study used publicly available, de-identified transcriptomic and genetic summary datasets (e.g., GEO, GTEx, and publicly released GWAS summary statistics). No new human participants were recruited, and no identifiable private information was accessed. Therefore, institutional review board approval and informed consent were not required for this work, in accordance with applicable guidelines for the use of publicly available de-identified data.

## Funding

This research was supported by the Suzhou Gusu Innovation Leading Talent Program (ZXL2022483 to Hang Ruan), Basic Research Program of Jiangsu (No. BK20255001) and the 10.13039/501100012246Priority Academic Program Development of Jiangsu Higher Education Institutions.

## CRediT authorship contribution statement

**Ziyi Chen:** Data curation, Software, Visualization, Writing – original draft, Writing – review & editing. **Jingjing Shi:** Data curation, Writing – review & editing. **Yusuo Meng:** Investigation, Methodology, Software. **Sidong Xiong:** Conceptualization, Resources, Supervision. **Hang Ruan:** Conceptualization, Methodology, Project administration, Resources, Validation, Writing – review & editing.

## Declaration of competing interest

The authors declare no competing interest.

## Data Availability

Data will be made available on request.
