# Supplementary material for: Systematic dysregulation of immune-related alternative polyadenylation in systemic lupus erythematosus contributes to patient stratification
Source: J Transl Autoimmun. 2026 Jun 5;12:100378. doi: 10.1016/j.jtauto.2026.100378 (PMC13260204; doi:10.1016/j.jtauto.2026.100378)
Supplement: Multimedia component 1 [file mmc1.docx]

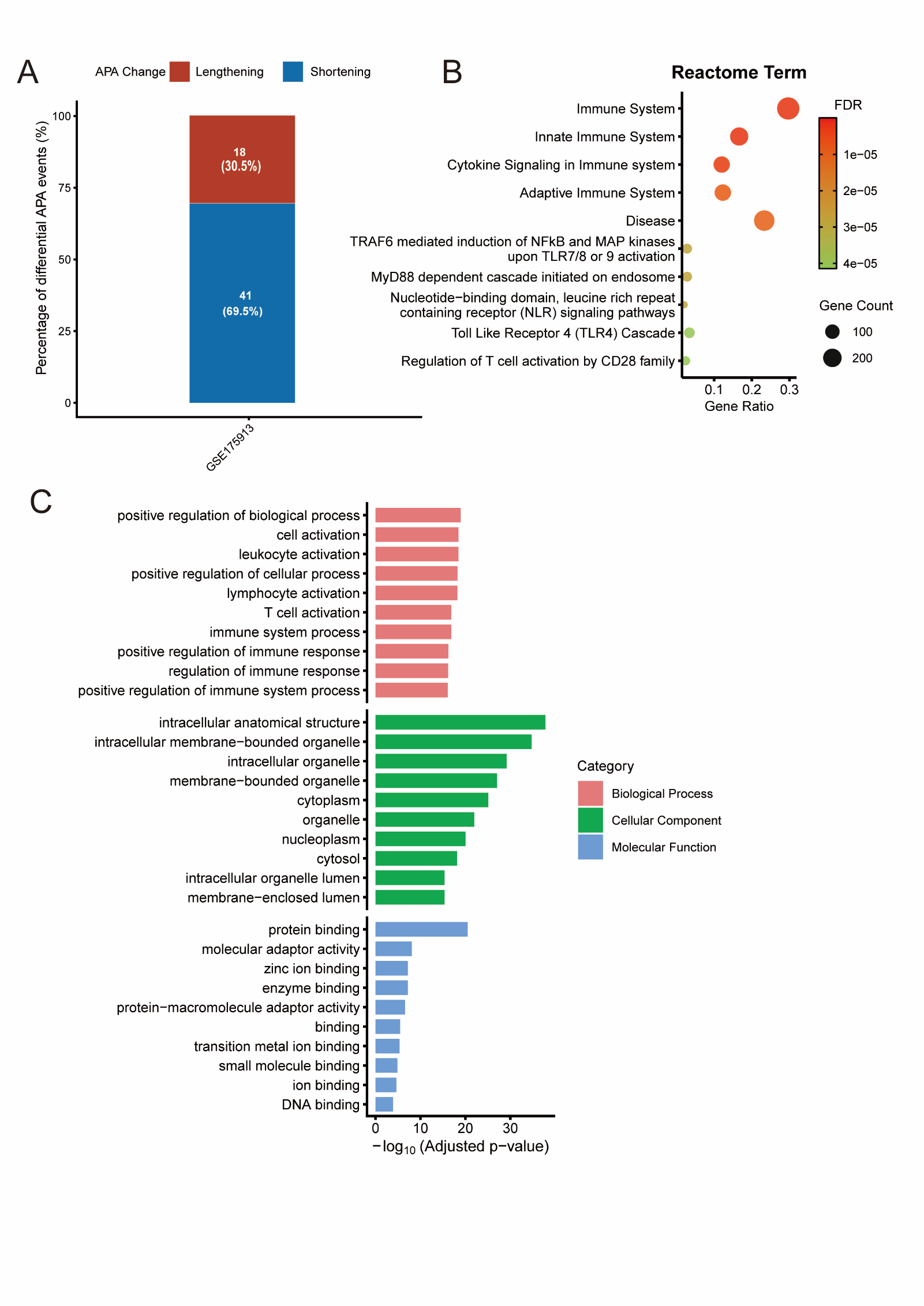


**Supplementary Figure 1** Supplementary results for the directional composition of differential APA events and functional enrichment of multi-cohort–consistent lengthening events.

(A) In the GSE175913 cohort derived from B cells, differentially expressed APA events were classified and statistically analyzed based on PDUI change direction. (B) Reactome pathway enrichment analysis of genes corresponding to multi-cohort–consistent “lengthening” differential APA events. The x-axis indicates the gene ratio; bubble size represents the number of enriched genes (gene count); color denotes the multiple-testing–adjusted significance level (FDR). (C) Gene Ontology (GO) enrichment analysis of the same gene set, showing significant terms across Biological Process (BP), Cellular Component (CC), and Molecular Function (MF). The x-axis denotes −log10(adjusted P value).


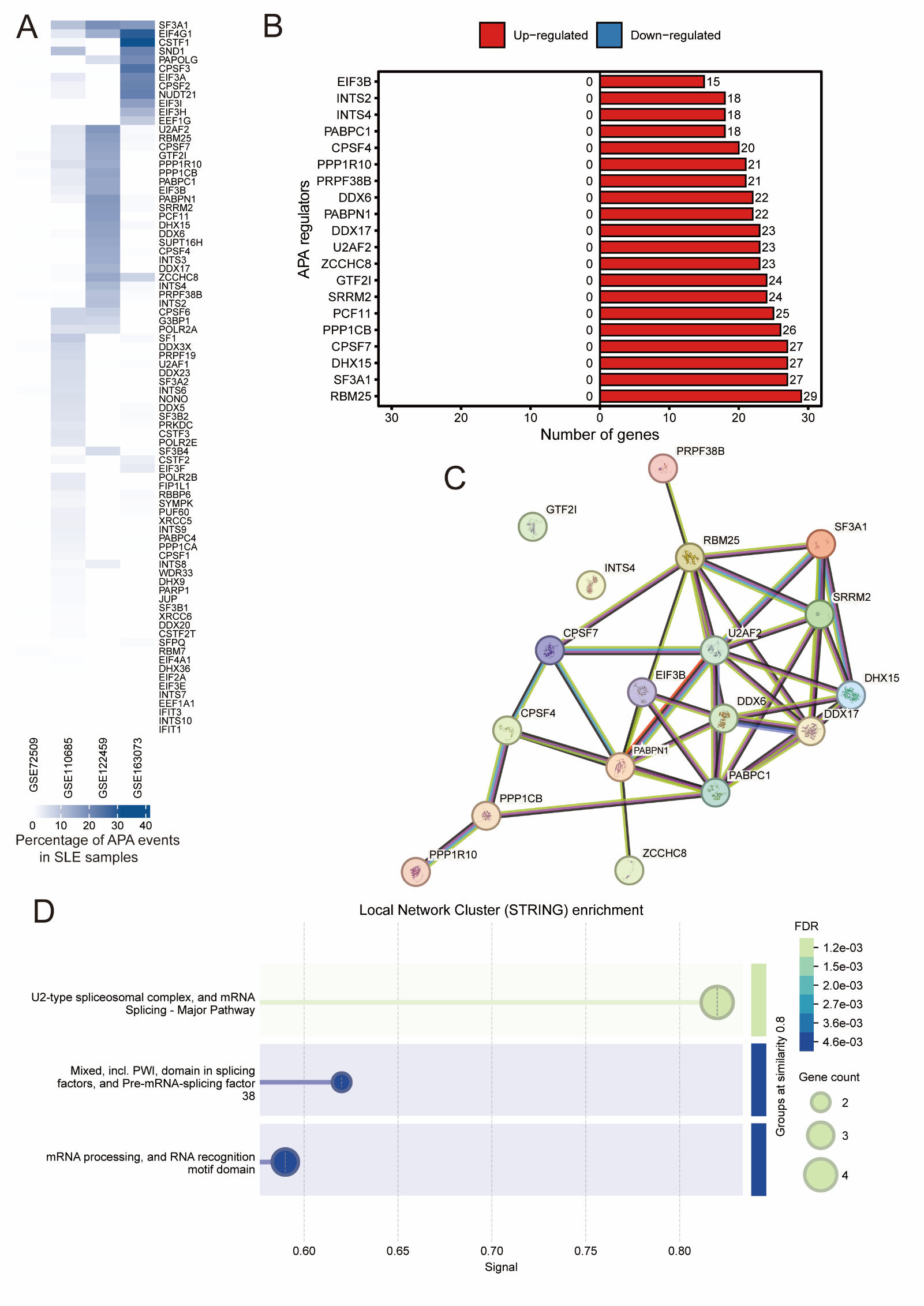


**Supplementary Figure 2** analyses of APA regulators, including event-association proportions, directionality of consistently associated genes, and PPI/functional module characterization.

(A) Heatmap showing, within each cohort and restricted to SLE samples, the proportion (%) of APA events that are significantly negatively associated with each APA regulator. Color intensity indicates the percentage of negatively associated events. (B) Summary of the numbers of target genes that are consistently associated with the key APA regulators across multiple cohorts, stratified by directionality. “Up” and “Down” correspond to genes upregulated or downregulated in SLE, respectively. The x-axis denotes gene counts, reflecting the putative regulatory coverage and directional preference of each regulator. (C) Protein–protein interaction (PPI) network of core APA regulators (e.g., derived from STRING). Nodes represent regulator proteins and edges indicate known or predicted interactions, illustrating the network architecture of key regulators within spliceosome- and 3′-end processing–related complexes. (D) Functional enrichment of local network clusters identified from the PPI network. The x-axis indicates enrichment signal strength (Signal); bubble size denotes gene count and color represents FDR.


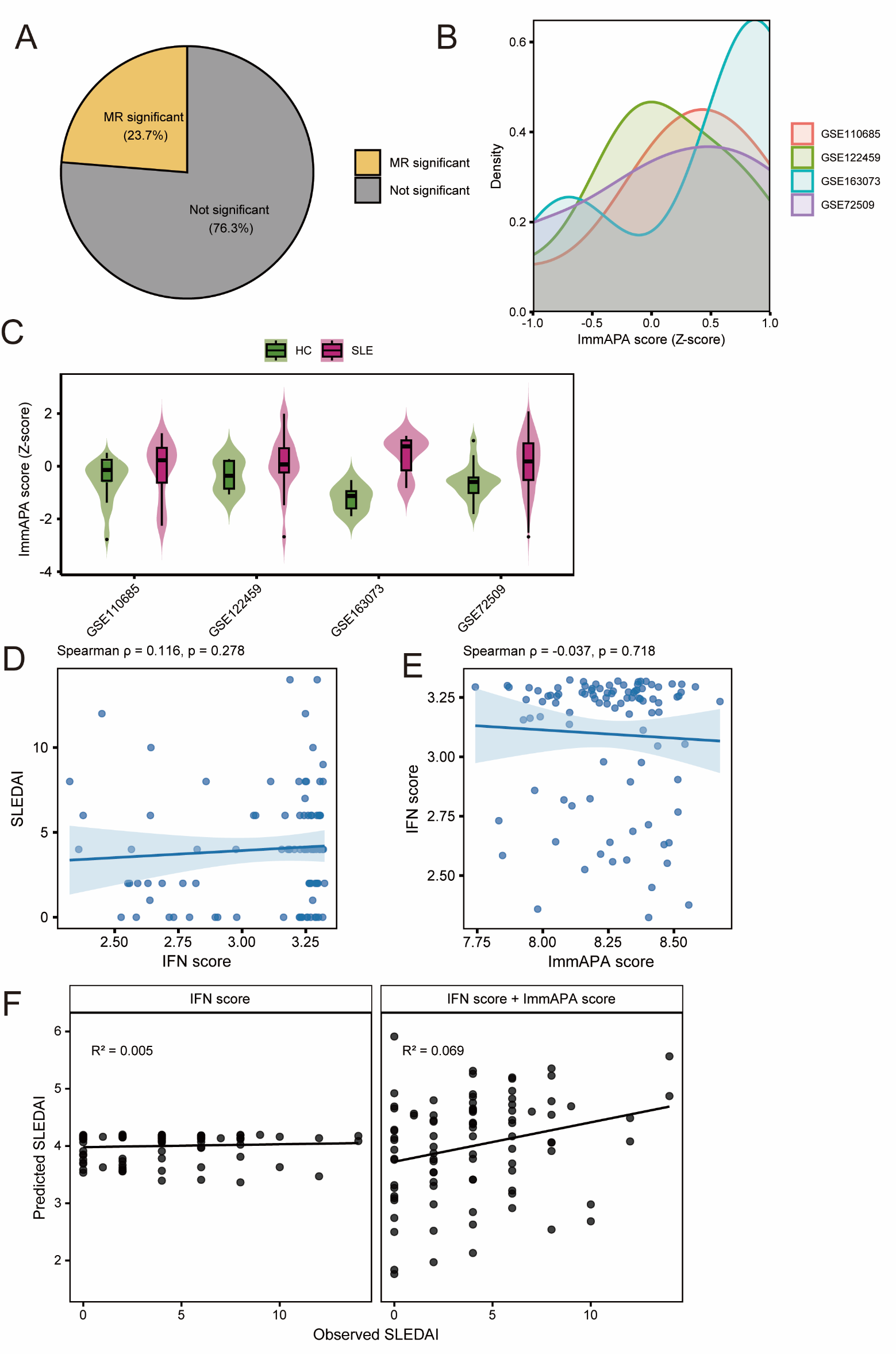


**Supplementary Figure 3** APA Score-Based Subtyping of SLE

(A) Pie chart showing the proportion of significant results in the Mendelian randomization (MR) analysis, including MR significant (23.7%) and Not significant (76.3%). (B) Density plots illustrate the distribution curves of ImmAPA Score (Z-score) across different datasets. (C) Violin plots compare ImmAPA Score (Z-score) distributions between healthy controls (HC, green) and SLE patients (pink) across various datasets. (D) Spearman correlation analysis reveals a correlation between SLEDAI scores and IFN scores (ρ = 0.116, p = 0.278). The scatterplot displays individual data points with a trendline and confidence intervals. (E) Spearman correlation analysis reveals a negative correlation between IFN scores and ImmAPA scores (ρ = -0.037, p = 0.718). The scatter plot displays individual data points with a trendline and confidence intervals. (F) Observed versus predicted SLEDAI values from nested linear regression models using IFN score alone or IFN score plus ImmAPA score. Each point represents one SLE patient. The fitted line indicates the relationship between observed and model-predicted SLEDAI. Compared with the IFN-only model, adding ImmAPA score improved the model fit and increased the explanatory performance for SLEDAI, suggesting that ImmAPA score provides complementary SLEDAI-related information beyond the traditional IFN score.


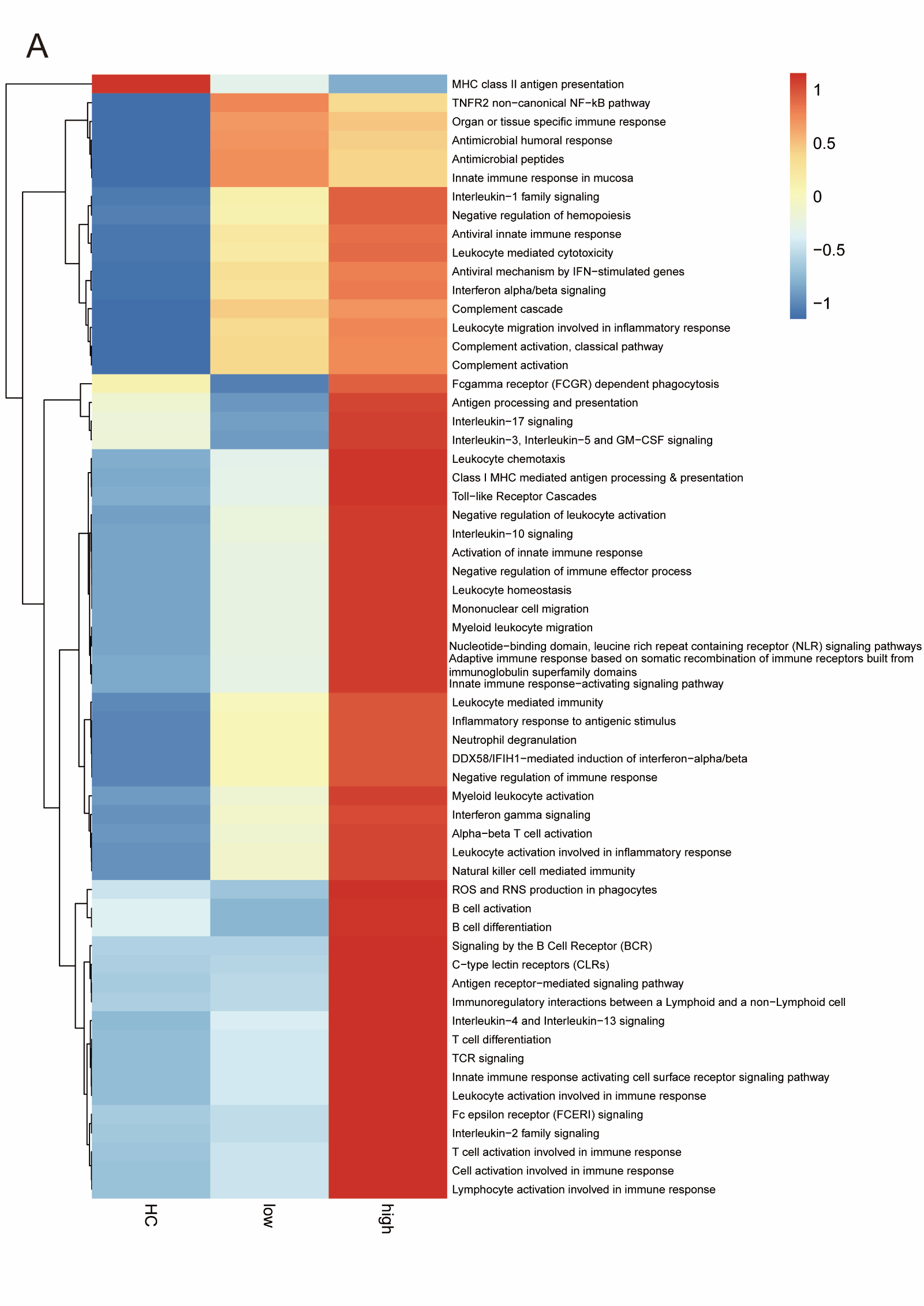


**Supplementary Figure 4** Activation Levels of Immune-Related Pathways in Different SLE Subgroups

(A) Heatmaps display the average pathway score (Z-score) for various immune-related pathways across healthy controls (HC), ImmAPA_low, and ImmAPA_high. Each value represents the average Z-score for that pathway across samples within each group. Rows indicate clustered pathways, while columns denote subgroups (HC, low, high). The color bar transitions from blue (-1, indicating low activation or negative regulation) to red (1, indicating high activation or positive regulation).


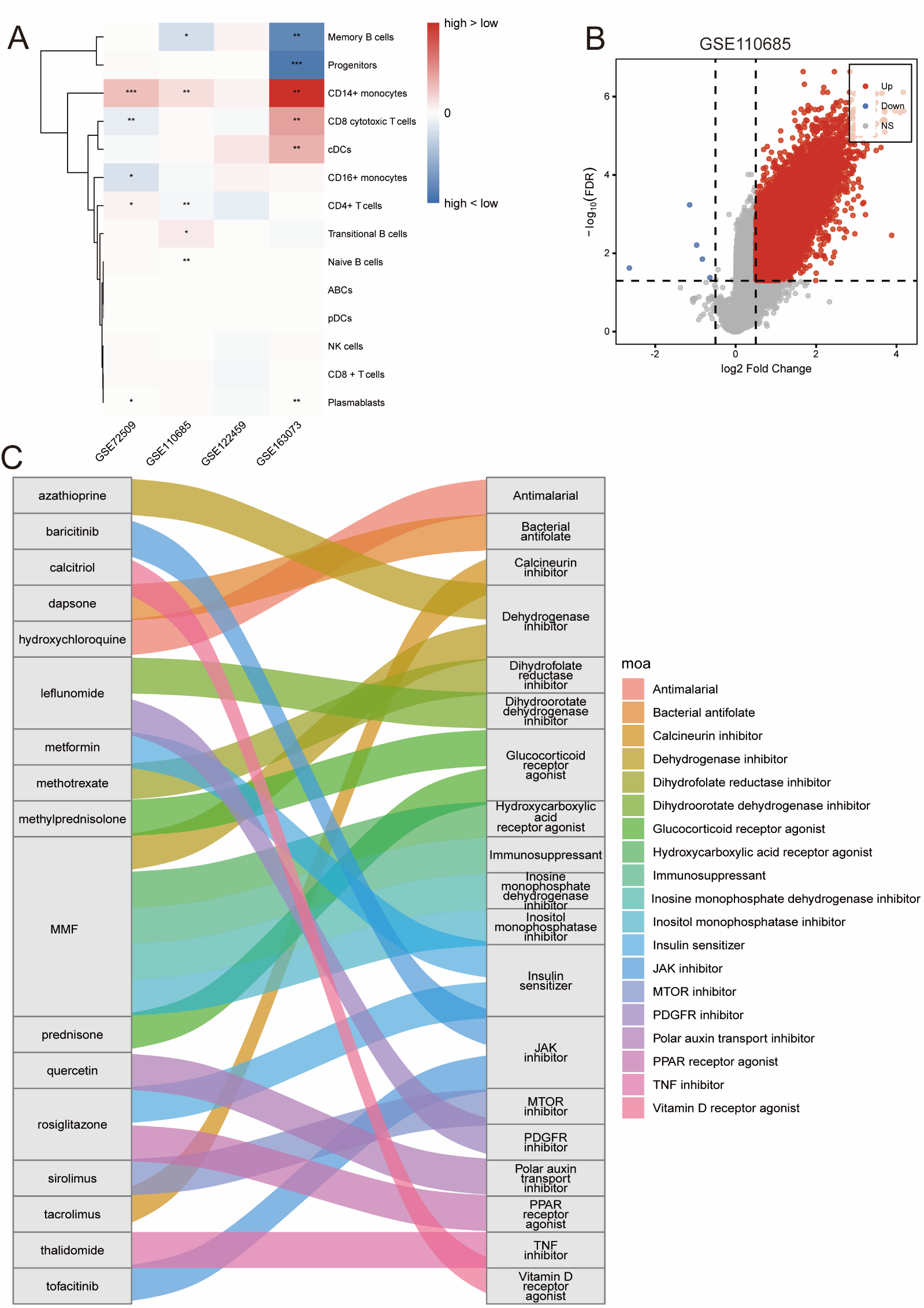


**Supplementary Figure 5** Additional analyses supporting ImmAPA-guided drug prioritization and MMF-related clinical stratification

(A) CIBERSORTx was used to estimate immune cell proportions in each cohort. The heatmap shows differences in inferred immune cell proportions between ImmAPA-high and ImmAPA-low groups. Values represent mean differences calculated as ImmAPA-high minus ImmAPA-low. Red indicates higher inferred cell proportions in ImmAPA-high, whereas blue indicates lower inferred cell proportions in ImmAPA-high. Asterisks indicate Wilcoxon rank-sum test significance after FDR correction. The results show that some immune cell populations, such as CD14 monocytes and memory B cells, differed between ImmAPA-high and ImmAPA-low groups in several cohorts, but these differences were not uniformly significant across all datasets. (B) Volcano plot of APA_high vs. APA_low differentially expressed genes in GSE72509: log2 fold change on the x-axis, −log10(FDR) on the y-axis; dashed line indicates screening threshold; red indicates upregulation, blue indicates downregulation, gray indicates no significant difference. (C) Sankey diagram showing candidate drugs and their corresponding mechanism of action (MOA) categories; drug names are listed on the left, MOA categories on the right, with lines indicating the primary MOA classification for each drug.
